# Investigation of the efficacy and safety of retinal inactivation as a treatment for amblyopia in cats

**DOI:** 10.3389/fnins.2023.1167007

**Published:** 2023-06-20

**Authors:** Mairin Hogan, Nadia R. DiCostanzo, Nathan A. Crowder, Ming-fai Fong, Kevin R. Duffy

**Affiliations:** ^1^Faculty of Health, Clinical Vision Science, Dalhousie University, Halifax, NS, Canada; ^2^Department of Psychology and Neuroscience, Dalhousie University, Halifax, NS, Canada; ^3^Department of Biomedical Engineering, Georgia Institute of Technology, Atlanta, GA, United States

**Keywords:** refractive error, ocular axial length, tetrodotoxin, retinal inactivation, visually-evoked potentials, amblyopia, plasticity, visual cortex

## Abstract

**Introduction:**

Deprivation of normal vision early in postnatal development elicits modifications of neural circuitry within the primary visual pathway that can cause a severe and intractable vision impairment (amblyopia). In cats, amblyopia is often modeled with monocular deprivation (MD), a procedure that involves temporarily closing the lids of one eye. Following long-term MD, brief inactivation of the dominant eye’s retina can promote recovery from the anatomical and physiological effects of MD. In consideration of retinal inactivation as a viable treatment for amblyopia it is imperative to compare its efficacy against conventional therapy, as well as assess the safety of its administration.

**Methods:**

In the current study we compared the respective efficacies of retinal inactivation and occlusion of the dominant eye (reverse occlusion) to elicit physiological recovery from a prior long-term MD in cats. Because deprivation of form vision has been associated with development of myopia, we also examined whether ocular axial length or refractive error were altered by a period of retinal inactivation.

**Results:**

The results of this study demonstrate that after a period of MD, inactivation of the dominant eye for up to 10 days elicited significant recovery of visually-evoked potentials that was superior to the recovery measured after a comparable duration of reverse occlusion. After monocular retinal inactivation, measurements of ocular axial length and refractive error were not significantly altered from their pre-inactivation values. The rate of body weight gain also was not changed during the period of inactivation, indicating that general well-being was not affected.

**Discussion:**

These results provide evidence that inactivation of the dominant eye after a period of amblyogenic rearing promotes better recovery than eye occlusion, and this recovery was achieved without development of form-deprivation myopia.

## Introduction

Unobstructed and concordant binocular vision plays an important role in development and function of neurons within the primary visual pathway. Disruption of early binocular vision by unilateral cataract, unequal refractive error between the eyes, or misalignment of the eyes can derail normal neural development and impair function of the affected eye. In research animals, this is perhaps best exemplified by simply closing the lids of one eye early in postnatal development, a procedure referred to as monocular deprivation (MD). Neural abnormalities in the primary visual pathway acquired from rearing with MD can precipitate a severe and intractable impairment of vision, called amblyopia, that is characterized by reduced spatial acuity (Wiesel and Hubel, 1963; Giffin and Mitchell, 1978) and loss of binocularity (Timney, 1983; Vorobyov et al., 2007). Neural modifications within the primary visual pathway are thought to seed abnormalities elsewhere, producing a cascade of dysfunctions that can include deficiencies in eye-hand coordination (O’Connor et al., 2010; Kelly et al., 2019), fine motor skill (Webber et al., 2016; Kelly et al., 2019), and reading skill (Kelly et al., 2015), all of which can limit opportunities and culminate in lowering self-esteem (Birch et al., 2019).

In humans, amblyopia is commonly treated with “patching,” a procedure that involves occluding the dominant eye to promote use and recovery of the weaker one. Notwithstanding its widespread use for centuries, patching therapy is hindered by limitations that can subvert good recovery outcomes for patients. Foremost on the list of limitations is the lack of compliance with prescribed patching, which in part derives from an associated social stigma with the therapy, as well as from the unavoidable disability that results from being forced to use only the impaired eye (Stewart et al., 2003; Holmes and Levi, 2018). Even following a high degree of patching compliance that yields good recovery, a considerable number of patients experience recurrence of amblyopia upon completion of patching therapy (The Pediatric Eye Disease Investigator Group, 2004; Bhola et al., 2006; Jia et al., 2022).

Neural abnormalities thought to represent the core pathology of deprivation amblyopia have recently been shown to resolve following a brief duration of monocular inactivation (MI) applied at ages beyond the critical period peak (Duffy et al., 2018; Fong et al., 2021). Brief inactivation of the dominant eye’s retinal ganglion cells with microinjection of tetrodotoxin (TTX), a voltage-gated sodium channel blocker, promoted recovery of neuron soma size in deprived layers of the dorsal lateral geniculate nucleus (dLGN) of cats, and also elicited recovery of deprived-eye visually-evoked potentials (VEPs) in cat and mouse primary visual cortex (V1; Duffy et al., 2018; Fong et al., 2021). In animal models of deprivation amblyopia, the gold standard treatment against which the efficacy of novel treatments is judged, is a procedure called reverse occlusion (RO) that is analogous to human full-time patching and involves opening the deprived eye and then closing the dominant one (Wiesel and Hubel, 1965; Blakemore and Van Sluyters, 1974). The first aim of the current study was to compare the extent of recovery from MD in animals subjected to MI of the dominant eye with the recovery elicited by a comparable duration of RO.

Of paramount importance in the consideration of MI as a treatment for amblyopia is demonstration of its safe application. In cats, MI does not alter the size or density of cells in the retinal ganglion cell layer, nor does it alter neurofilament labeling or myelin staining in the optic nerve serving the inactivated eye (DiCostanzo et al., 2020). Furthermore, VEPs recorded from V1 of monkeys and cats subjected to brief MI recover to baseline levels after the effects of inactivation wear off (Foeller and Tychsen, 2019; DiCostanzo et al., 2020; Fong et al., 2021), suggestive that the impact of inactivation on the retina is transient and reversible.

In humans, excessive patching therapy for amblyopia during early development has been linked to high myopia (Muñoz and Capo, 1995), and unilateral eye closure by ptosis can also associate with the development of myopia in the deprived eye (O’Leary and Millodot, 1979; Hoyt et al., 1981; Huo et al., 2012). In animal models, closure of the lids of one eye in neonatal macaque monkeys can produce axial myopia by elongating the deprived eye (Wiesel and Raviola, 1977; Smith et al., 1987). Similar results are observed in visually deprived tree shrews (Sherman et al., 1977), and chicks (Wallman et al., 1978). Results in cats appear less clear, with some studies showing that long-term MD produces axial elongation and myopia in the deprived eye (Wilson and Sherman, 1977; Kirby et al., 1982), while others have reported that these effects are unreliable (Gollender and Thorn, 1979; Nathan et al., 1984). Unlike monocular lid closure that deprives form vision in the affected eye, MI eliminates impulses from retinal ganglion cells. That MI can exhibit greater efficacy to elicit neural changes in the dLGN compared to lid closure (Duffy et al., 2018, 2023), raises the possibility that ocular refractive error and axial length may be altered by MI. If MI modifies either of these characteristics, it could produce imbalanced focusing ability between the eyes in patients treated for amblyopia. Therefore, the second objective of the current study was to examine whether brief MI produced an imbalance in either ocular refractive error or axial length.

## Materials and methods

### Animals

Fifteen animals were examined in this study. All were reared from birth in a closed breeding colony at Dalhousie University. All animals were part of other experiments that examined the efficacy of fellow-eye inactivation to promote recovery from the effects of MD (Duffy et al., 2018; DiCostanzo et al., 2020; Fong et al., 2021); therefore, there is some variability in the visual experience of animals in this study. The complete rearing history of animals is presented in Table 1. We examined groups of animals that either (1) received 10 days of retinal inactivation after being raised with normal binocular vision (*n* = 5; three females, two males), or (2) received up to 10 days of retinal inactivation after a period of MD (*n* = 6; three females, three males). To assess the comparative efficacy of MI to promote recovery from MD, we also examined VEPs in a group of animals subjected to RO after a period of MD (*n* = 4; two females, two males). For all animals in this study, measurements from the non-inactivated eye acted as a control. All procedures in this study were approved by the standing committee overseeing animal care and ethics at Dalhousie University, and they adhered to use guidelines directed by the Canadian Council on Animal Care.

**Table 1:** Animal rearing conditions.

	Rearing manipulations				
Animal groups	Normal	MD	BV	Inactivation	BV
MD + Inactivation					
•C474 (f)	P0–P30	P30–P51	P51–P65	P65–P75	P75–P95
•C476 (f)	P0–P30	P30–P51	P51–P58	P58–P66	P66–P78
•⋄C479 (f)	P0–P30	P30–P51	P51–P58	P58–P80	P80–P100
⋄C478 (m)	P0–P30	P30–P51	P51–P79	P79–P89	P89–P96
⋄C482 (m)	P0–P30	P30–P51	P51–P107	P107–P117	P117–P131
⋄C484 (m)	P0–P30	P30–P51	P51–P68	P72–P80	P76–P96
⋄C485 (f)	P0–P70	-	-	P70–P80	-
⋄C486 (m)	P0–P70	-	-	P70–P80	P80–P90
⋄C491 (f)	P0–P70	-	-	P70–P80	-
⋄C492 (f)	P0–P42	-	-	P42–P52	-
⋄C493 (m)	P0–P70	-	-	P70–P80	P80–P95
	**Normal**	**MD**	**BV**	**RO**	**BV**
MD + RO
•C477 (m)	P0–P30	P30–P49	P49–P56	P56–P66	P66–P68
•C480 (f)	P0–P30	P30–P49	-	P49–P59	P59–P63
•C481 (m)	P0–P30	P30–P49	-	P49–P59	P59–P63
•C483 (f)	P0–P30	P30–P49	P49–P56	P56–P66	P66–P70

**Figure 1:**
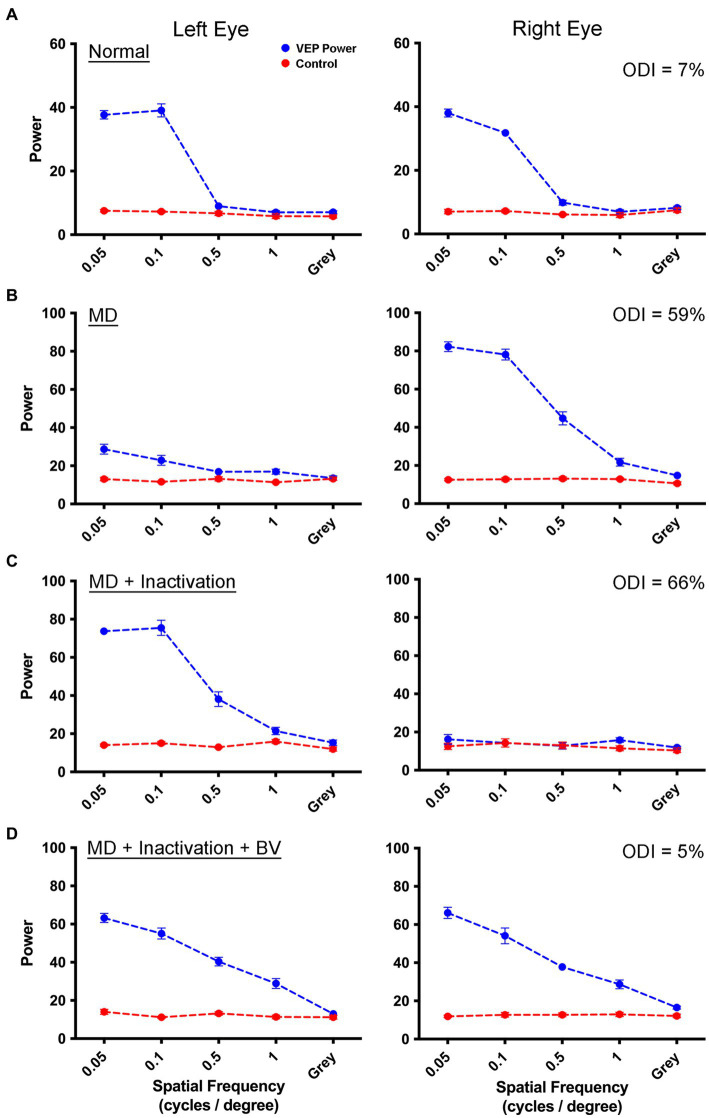
Measurement of VEPs from the left V1 elicited by separate stimulation of the left and right eye in a monocularly deprived animal that received fellow eye inactivation. For each graph, spatial frequency is plotted on the abscissa, and the summed power from the Fourier analysis is plotted on the ordinate. The blue trace represents the sum of visually-evoked power, while the red trace shows the non-visual baseline power. Visually-evoked power elicited by a gray screen served as a control, and should be about equal for the blue and red traces. Data are shown for an example animal (C479) in which VEP power from the left and right eye were balanced (blue traces) prior to any visual manipulation **(A)**. Following 3 weeks of left eye MD, there was a strong shift in ocular dominance with VEPs obviously attenuated for the deprived eye, and potentiated for the fellow non-deprived eye **(B)**. Inactivation of the non-deprived (right) eye for 8 days produced a recovery of VEPs in the originally deprived eye, while VEPs serving the inactivated eye remained at baseline levels due to the continued effect of TTX **(C)**. Following a washout period of 2 weeks, VEPs serving the inactivated eye were restored and in balance with those from the originally deprived eye **(D)**. For all animals in this study, VEPs exhibited lower power at the youngest age examined **(A)** compared to older animals **(D)**. It is unclear what produced this difference but it could be the result of a progressive maturation of V1 from 4 weeks of age to about 12 weeks of age. The percentage difference between VEPs measured between the two eyes across all spatial frequencies (ODI) for each rearing condition is displayed in the upper right corner of the right eye VEP graphs.

**Figure 2:**
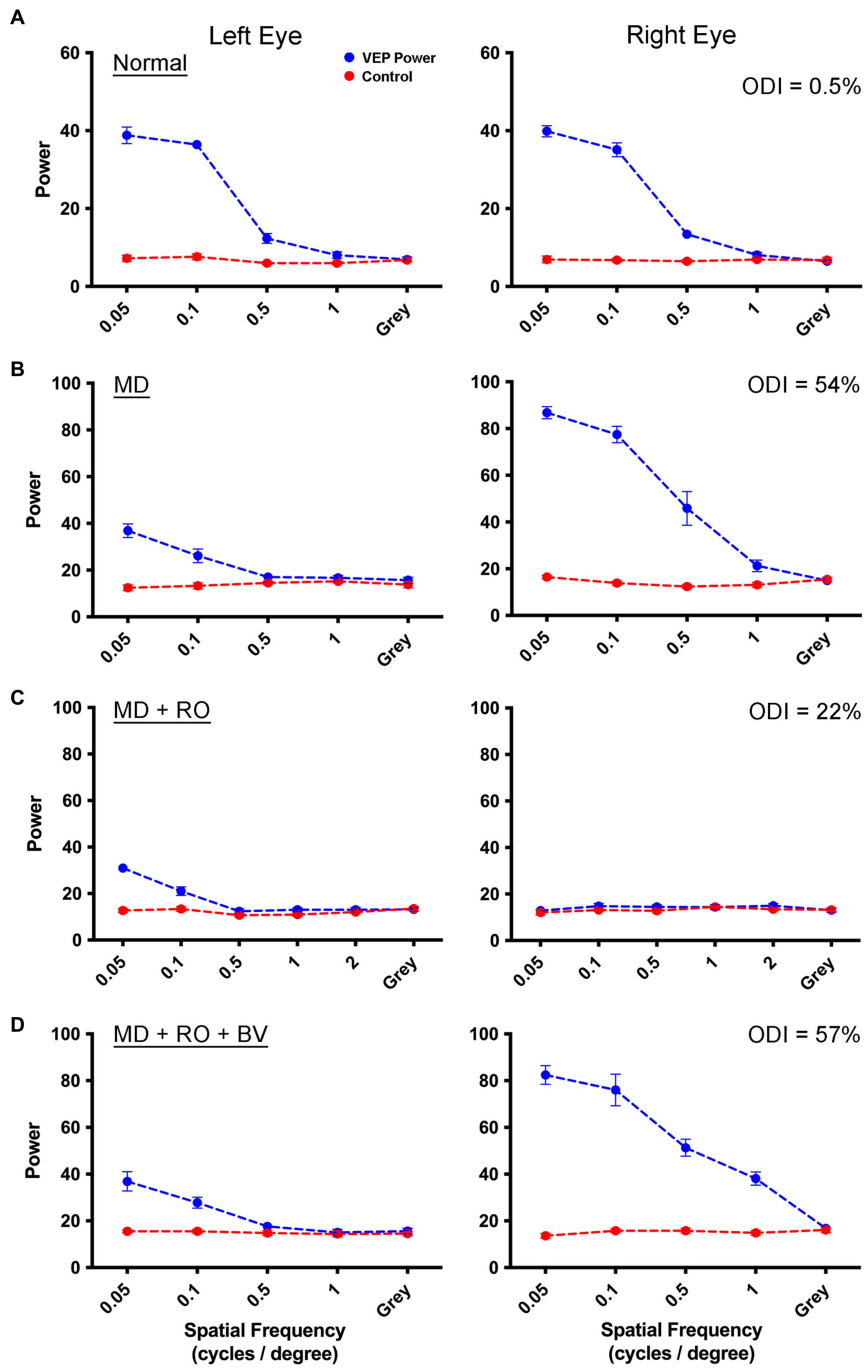
Measurement of VEPs from left V1 elicited by separate stimulation of the left and right eye in a monocularly deprived animal that received RO. Symbol and graph details are the same as those for Figure 1. Data are shown for an example animal (C481) in which VEP power from the left and right eye are balanced prior to any visual manipulation **(A)**. VEPs measured after 3 weeks of left-eye MD were attenuated for the deprived eye, but were potentiated for the fellow non-deprived eye indicative of a substantial shift in ocular dominance **(B)**. VEPs measured from the originally deprived eye did not increase appreciably after RO **(C)**, and measurements from originally non-deprived eye were attenuated to baseline because recordings were made while the eye was still closed. After the eye was opened and binocular vision was provided for 4 days, VEPs measured from the originally deprived eye remained diminished, while VEPs elicited from the eye that received RO were restored to their potentiated state before the RO was imposed **(D)**. This indicated that 10 days of RO imposed after 3 weeks of MD was insufficient to produce a substantial recovery of the deprived eye. The percentage difference between VEPs measured between the two eyes (ODI) for each rearing condition is displayed in the upper right corner of the right eye VEP graphs.

**Figure 3:**
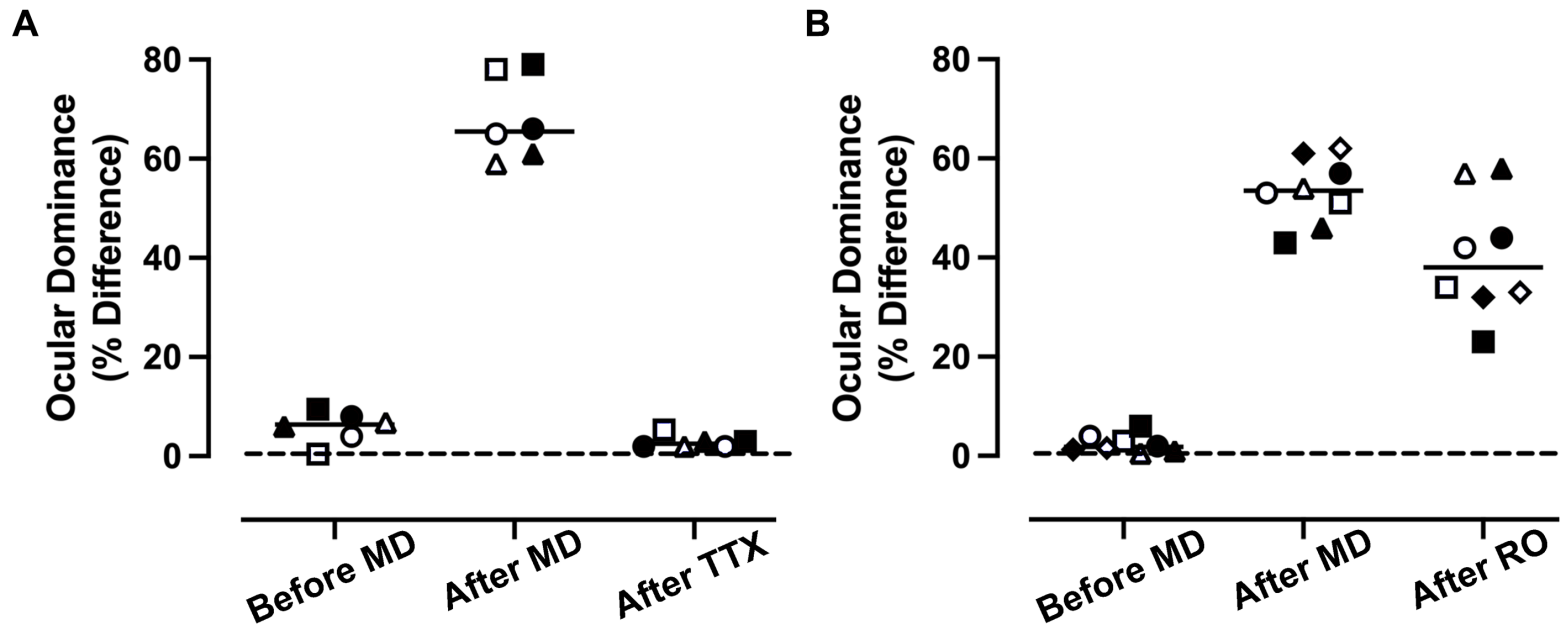
Comparison of the recovery promoted by MI or RO following 3 weeks of MD. Recovery was assessed with a calculation of ocular dominance that involved calculating the percentage difference in summed VEP power across all spatial frequencies that were presented separately to the left and right eye (see the section Materials and methods). For the MI group (*n* = 3 animals), balanced ocular dominance measured before MD was altered significantly after 3 weeks because VEP power was reduced by 68% in the deprived eye relative to the non-deprived eye **(A)**. After 3 weeks of MD, animals that had their fellow eye inactivated showed a recovery of normal ocular dominance in which summed VEP power was not different between the eyes. The comparison group of RO animals (*n* = 4) also showed a shift in ocular dominance so that VEP power for the deprived eye was reduced by 54%. Ten days of RO after the period of MD did not restore normal ocular dominance **(B)**. Open and solid symbols represent left and right V1, respectively. Symbol shapes represent different animals.

**Figure 4:**
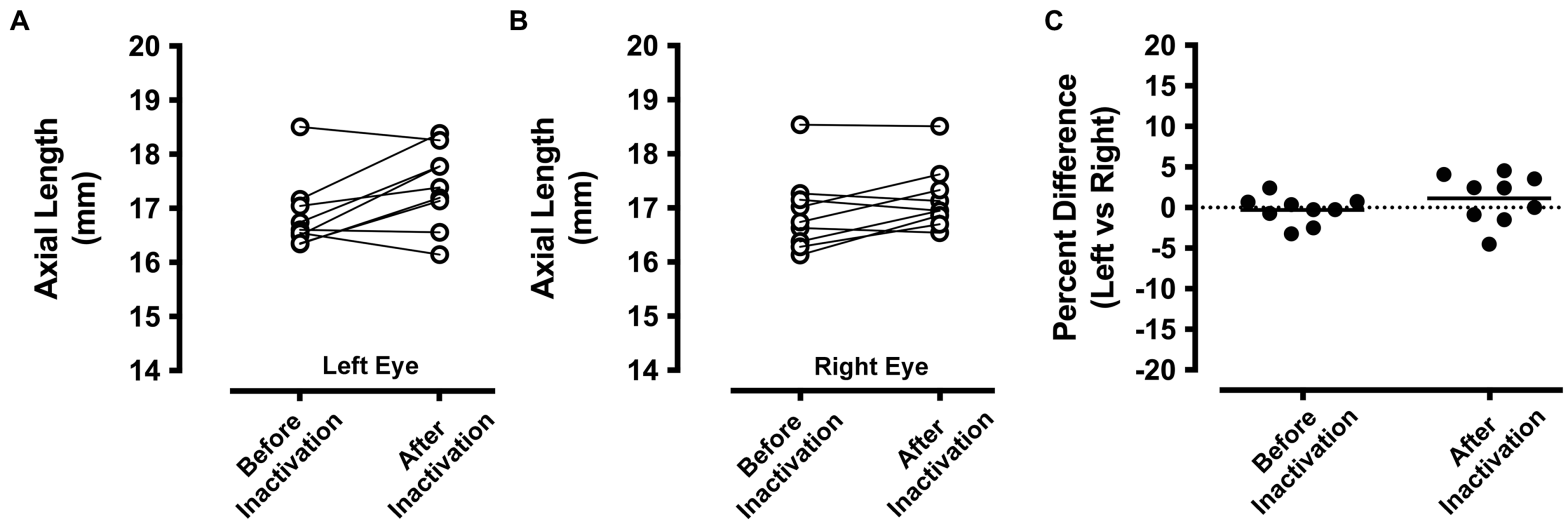
Ocular axial length measurements before and after inactivation of the right eye (*n* = 9 animals). A-scan measurements of the left eye that were obtained before MI were comparable to those taken after the period of inactivation **(A)**. A similar result was observed for the right eye, which showed before inactivation axial length measurements that were similar to those taken after inactivation **(B)**. Axial length measurements from the left and right eye were compared before and after inactivation by calculating the percentage difference between the eyes. With this metric, a value of zero indicates no difference between the eyes. Measurements from the left and right eye were close to zero both before and also after inactivation indicating that inactivation of the right eye did not produce an elongation or shortening of ocular axial length relative to the left eye **(C)**.

**Figure 5:**
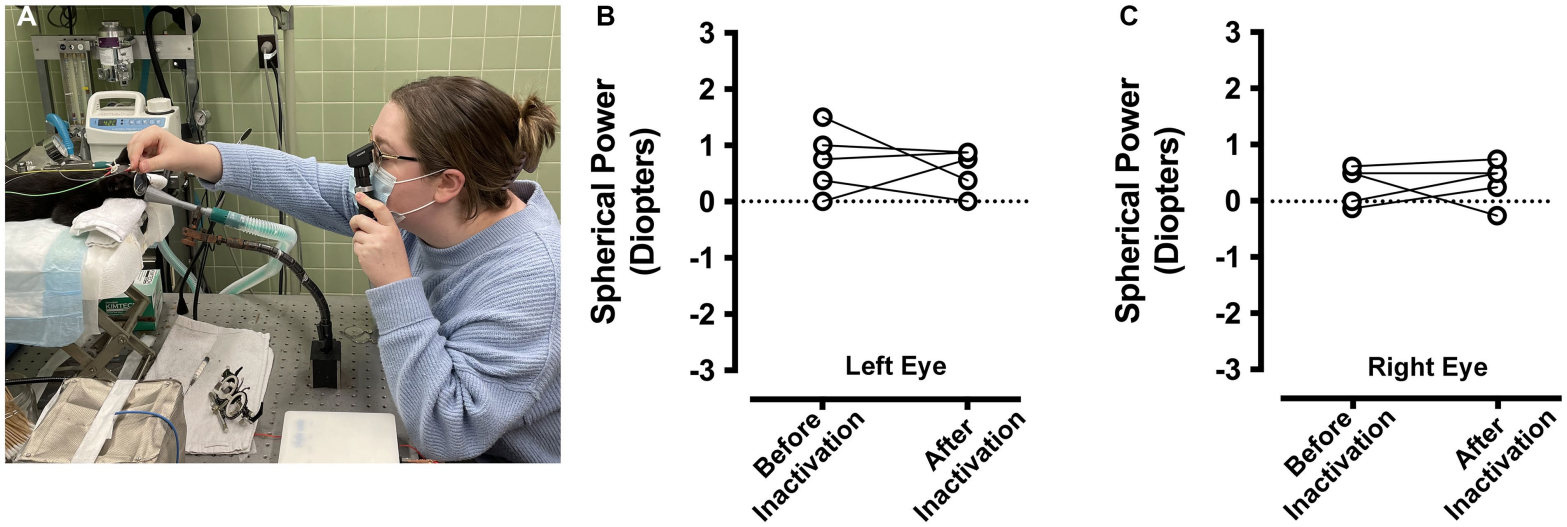
Measurement of ocular refractive error before and after right eye inactivation (*n* = 5 animals). Measurements of ocular refractive error were made by certified orthoptists using the loose lens retinoscopy method **(A)**. For the left eye, refractive error measured before inactivation was comparable to measurements after inactivation **(B)**. Refractive error measured for the right eye before inactivation were similar to those taken after inactivation **(C)**. For the purpose of capturing an image of the refraction process, the room lights were kept on for the photograph in **(A)**.

• Represents animals from which VEPs were recorded for Figures 1–3. ⋄ represents animals from which measurements of axial length and refractive error were obtained (also see Table 2) for Figures 4, 5. BV refers to binocular vision; RO refers to reverse occlusion. C479 received 5 days of BV after 2 days of inactivation, and then received 8 more days of inactivation. C476 and C484 received 8 days of inactivation. Male (m) and female (f) animals are indicated beside animal number.

### Body weight

In a subset of animals subjected to MI, we measured body weight throughout the duration of their involvement in the study. Body weight was tracked as a proxy for systemic toxicity and well-being during MI. Measurements of body weight were made using an Ohaus compact scale (Fisher Scientific, Canada) that was calibrated to have sensitivity from 1 g to 5.2 kg.

### Monocular deprivation

Some animals in this study were reared with MD to produce amblyopia before receiving up to 10 days of fellow-eye inactivation or reverse occlusion. Monocular deprivation surgery on the left eye was performed under general anesthesia using 3–4% isoflurane in oxygen. The upper and lower palpebral conjunctivae of the left eye were sutured closed using vicryl suture material, which was followed by closure of the left eyelids with silk suture material. The lid closure procedure lasted about 15 min after which animals were administered subcutaneous ketoprofen (2 mg/kg; CDMV, Canada) as a means of postoperative analgesia, and were given topical ophthalmic Alcaine (proparacaine hydrochloride; CDMV) for local anesthesia. A broad-spectrum topical antibiotic (1% Chloromycetin; CDMV) was applied to the eyelids to mitigate infection. After the period of MD, animals had their deprived eye opened under general anesthesia (3–4% isoflurane), then either had their dominant eye inactivated (MI) or had their dominant eye closed (RO) for 10 days. Some animals had a period of binocular vision before MI or RO was initiated.

### Retinal inactivation

Animals subjected to MI were anesthetized with 3–4% isoflurane and the right eye was administered an intravitreal injection of TTX (ab120055; Abcam, United States) solubilized in citrate buffer at 3 mM. Animals that received MD had their deprived eye opened before inactivation of the fellow eye. For each animal, dosage was scaled according to eye size (Thorn et al., 1976). We administered 0.5 μL of TTX per mm of vitreous chamber length. This dosage blocks action potentials of affected cells without obstructing critical cellular functions such as fast axoplasmic transport (Ochs and Hollingsworth, 1971). Injections were administered through a small puncture made in the sclera located at the pars plana using a sterile 30-gage needle. Using a surgical microscope, the measured volume of TTX solution was dispensed into the vitreous chamber using a sterilized Hamilton syringe (Hamilton Company, United States) with a 30-gage needle (point style 4) that was positioned through the original puncture and about 5–10 mm into the chamber angled away from the lens. The total volume of TTX was dispensed slowly, and when complete the needle was held in place for about a minute before it was retracted. Following intraocular injection, topical antibiotic (1% Chloromycetin) and anesthetic (Alcaine) were applied to the eye to prevent post-injection complications. To achieve the desired duration of inactivation, animals received one injection of TTX every 48 h, and for each injection, the original puncture site was used to avoid having to make another hole. A single dose of TTX administered intravitreally eliminates visual responses for at least 48 h (Wong-Riley and Riley, 1983; Stryker and Harris, 1986; Linden et al., 2009; Fong et al., 2016). During the period of inactivation, we employed basic assessments of visual behavior to confirm inactivation. We verified the absence of a pupillary light reflex as well as the lack of visuomotor behaviors such as visual placing, visual startle, and the ability to track a moving laser spot. These assessments were made while vision in the non-injected eye was briefly occluded with an opaque contact lens. In all cases, retinal inactivation (or RO) was performed on the right eye. Therefore, measurements made in this study were not performed blind to which eye was inactivated or subjected to occlusion.

### Visually-evoked potentials

In preparation for measurement of VEPs, animals were initially anesthetized with 3% isoflurane in oxygen that was reduced to between 1 and 1.5% during recordings. Supplemental sedation during recordings was provided with acepromazine (0.06–0.1 mg/kg) and butorphanol (0.1–0.2 mg/kg; I.M.). Hair on the head was trimmed and a disposable razor was used to shave parts of the scalp where recording sites were located. Two recording sites were positioned approximately 2–8 mm posterior and 1–4 mm lateral to either side of interaural zero over the presumptive location of right and left V1, and a third site over the midline of the frontal lobes that acted as a reference. Electrode sites were abraded with Nuprep EEG skin preparation gel (bio-medical, MI, United States), and were then further cleaned with alcohol pads. Reusable 10 mm gold cup Grass electrodes (FS-E5GH-48; bio-medical) were secured to each recording site using Ten20 EEG conductive paste (bio-medical, United States) that was applied to the scalp. Impedance of the recording electrode was measured in relation to the reference electrode to ensure values were below 5 kΩ. Electrophysiological signals were amplified and digitized with an Intan headstage (RHD2132; 20 kHz sampling frequency), then recorded using an Open Ephys acquisition board and GUI software (Open Ephys, United States). Visual stimuli were programmed in MatLab using the Psychophysics Toolbox extension (Brainard, 1997; Pelli, 1997), and presented on an LCD monitor (Dell 210-AMSR; 25″ display, 240 Hz refresh, 1,920 × 1,080 pixels) at a viewing distance of 70 cm. Steady state VEPs were elicited with full contrast square wave gratings with a 2 Hz contrast reversal frequency (Bonds, 1984; Pang and Bonds, 1991; Norcia et al., 2015). Gratings of different spatial frequencies (0.05, 0.1, 0.5, and 1 cycles/degree) or a blank gray screen were presented in random order for 20 s each, with a blank gray screen also displayed during the 2 s interstimulus interval. Each stimulus was presented for at least six repetitions. The viewing eye was tested in isolation by placing a black occluder in front of other eye during recording. The eyes were kept open with small specula, and were frequently lubricated with hydrating drops. Recording sessions lasted about 45 min and animal behavior was observed for at least 1 h post-recording to ensure a complete recovery. The raw electroencephalogram was imported to MatLab where it was high-pass filtered above 1 Hz, then subjected to Fourier analysis (Bach and Meign, 1999; Norcia et al., 2015). The magnitude of VEPs was calculated as the sum of power at the stimulus fundamental frequency plus six additional harmonics (2, 4, 6, 8, 10, 12, and 14 Hz). Baseline nonvisual activity was calculated as the sum of power at frequencies just offset from the visual response (2.45, 4.45, 6.45, 8.45, 10.45, 12.45, and 14.45 Hz).

### Refraction

Measurements of refractive error were obtained by objective retinoscopy. Retinoscopy was performed by certified ophthalmic care practitioners who are trained in retinoscopy. Following the initial assessment, a second clinician independently verified measurements. The Keeler Professional 3.6v Combi Retinoscope (Keeler, Halma plc, Malvern, PA, United States) was used to obtain measurements. Animals were anesthetized with 2–3% isoflurane with their body placed in a prone position and their head facing forward. Both eyes were unobstructed and positioned toward distance fixation. Specula were used to keep the eyes open during the procedure. Lubricating drops were applied frequently to each eye as needed. Loose-lens refraction was performed in a darkened room. There was no preference as to which eye was refracted first. A streak retinoscope was used to sweep across the pupil, observing the “with” or “against” motion of the beam, which was rotated 360 degrees until a first meridian was located. Stronger or weaker lenses, available in 0.25 Diopter (D) increments, were alternated as needed until neutralization was achieved. The retinoscope light beam then scoped the other meridians of the eye. If neutralization was not located at another angle, stronger and weaker lenses would be alternated at this new angle until neutralization was reached. The lens strength required at each angle to produce neutralization was recorded prior to removing the working distance. A pair of trial lens frames, etched with axial calibration scales, were held in front of the animal’s eyes to confirm the axis of astigmatism when needed. A loose-lens objective refraction was then completed for the other eye following the steps described above. Measurements were recorded as the spherical equivalent for refractive errors with less than 0.75 D of astigmatism.

### Ocular axial length

A-scan ultrasonic measurements of ocular axial length were made on anesthetized animals using an Accutome A-Scan Connect (Keeler United States, Malvern, PA). One drop of 10% phenylephrine hydrochloride was administered to each eye so that the nictitating membrane did not obstruct the cornea during measurements. Axial length measurements were made from animals after their refractive error was determined. At each session, five series of readings were independently obtained from each eye, and the average of the five readings was recorded as the axial length for the eye being measured. Measurements were made with an A-scan biometry probe using the applanation technique that involved gently placing the ultrasound probe directly on the corneal surface. All axial length measurements were made with the instrument set to phakic, contact, and automatic mode with the threshold set to 67 db.

### Statistical analyses

To assess possible differences in VEP power across experimental conditions, an ocular dominance index (ODI) was calculated to determine the percentage difference in VEP power between the left and right eye. The sum of VEP power measurements for all spatial frequencies presented to the non-dominant eye was subtracted from the sum of VEP power for the dominant eye, and this difference was divided by the sum of VEP power for the dominant eye. This produced a single number, calculated independently for each hemisphere that was expressed as the percentage difference between the eyes. For this calculation, the dominant eye was defined as the one with the highest summed VEP power. A repeated measures one-way ANOVA using Šídák multiple comparisons tests was employed to investigate the extent of recovery within the MI and RO groups. Comparison of the recovery achieved after MI and RO was achieved with an unpaired *t*-test. VEP measurements from left and right V1 were treated as single observations, and significance was set at 0.05. A repeated measures one-way ANOVA was also employed to compare measurements of refractive error before and after inactivation for the left and right eyes. Statistical comparison of before and after inactivation measurements for ocular axial length, and body weight were achieved using a two-tailed paired *t*-test with significance set at 0.05.

## Results

### Recovery of VEPs: MI vs. RO

We started our investigation by comparing the efficacy of MI with that of RO as a means to promote physiological recovery from the effects of a 3-week MD started at postnatal day 30, the critical period peak. Measurement of VEPs from V1 before MD showed balanced VEP power profiles between the left and right eyes (Figure 1A). Following 3 weeks of left eye MD, a clear imbalance developed between the eyes such that the deprived eye showed a substantial reduction in VEP power compared to the non-deprived eye that exhibited a potentiated response (Figure 1B). Inactivation of the dominant (right) eye for 8 days in this animal reduced VEP power in the right eye to non-visual baseline levels, but VEPs measured from the originally deprived eye exhibited a marked recovery, and even a potentiation, from the diminishment of responses produced by the prior MD (Figure 1C). Recovery of the originally deprived eye was sustained when inactivation of the fellow eye wore off, and VEPs serving the inactivated eye were restored and in balance with those measured from the other eye (Figure 1D). That VEPs in the inactivated eye were restored following a TTX washout period implies that there were no lasting detrimental effects of the inactivation on cortical responses.

We next examined whether the recovery achieved with MI was different from that obtained after a comparable duration of RO. This involved opening the initially deprived eye after the period of MD then closing the eyelids of the non-deprived eye, which is a procedure commonly used in animal models to mimic human patching therapy. Measurements collected from an example animal before imposing the MD showed VEPs that were balanced between the left and right eye in an example animal (Figure 2A). Following 3 weeks of MD to the left eye starting at postnatal day 30, VEP power serving the deprived eye was sharply attenuated while those serving the non-deprived eye exhibited an apparent potentiation (Figure 2B). Closure of the dominant (right) eye for 10 days did not stimulate recovery of VEP power measured from the originally deprived eye (Figure 2C). Data in Figure 2C are shown while the reverse occluded eye remained closed, and VEPs were consequently reduced to non-visual baseline levels. Following relief from RO and provision of a period of binocular vision, VEP power for the originally deprived eye remained attenuated in comparison to those from the fellow eye that remained in a potentiated state (Figure 2D). These results indicate that negligible recovery had occurred as a result of the 10-day period of RO.

As a way of comparing the efficacy of MI and RO to promote recovery, an ocular dominance index was calculated using VEP power measured for each eye separately (see the section Materials and methods). This permitted measurement of the percentage difference between the eyes across the three rearing conditions that were examined for the MI (Figure 3A) and RO (Figure 3B) groups, and for the left (open symbols) and right (solid symbols) visual cortex. The inactivation group showed a significant difference between rearing conditions as determined by a repeated measures ANOVA [*F*(2,5) = [288], *p* < 0.001; Figure 3A]. Measurements of VEP power from the normal, before MD, condition showed virtually no difference between the left and right eye as indicated by the near zero ocular dominance calculation. After 3 weeks of MD, there was a shift in ocular dominance so that VEP power was reduced in the deprived eye by an average of 65%, and this was significantly different from the normal condition before MD (Šídák’s multiple comparisons test; *p* < 0.001). When MD was followed by 10 days of MI and subsequent binocular vision, VEP power between the eyes was balanced and significantly different from after MD (Šídák’s multiple comparisons test; *p* < 0.001), but was not different from the normal condition before MD (Šídák’s multiple comparisons test; *p* = 0.38). The RO group likewise showed a significant difference between rearing conditions as determined by a repeated measures ANOVA [*F*(2,7) = [73.62], *p* < 0.001; Figure 3B]. At postnatal day 30 before imposing 3 weeks of MD, animals showed little difference between VEP power elicited by separate stimulation of each eye, indicating balance between the eyes. After MD, there was a shift in ocular dominance so that VEP power in the deprived eye was reduced by an average of 54% (Figure 3B), and this imbalance was significantly different from before MD (Šídák’s multiple comparisons test; *p* < 0.001). When MD was followed by 10 days of RO and subsequent binocular vision, the originally deprived eye VEP power was reduced to 40% in comparison to the fellow eye (Figure 3B), and this was still significantly different from the before MD condition (Šídák’s multiple comparisons test; *p* < 0.001). For RO animals, comparison of the after MD and after RO conditions revealed no statistical difference (Šídák’s multiple comparisons test; *p* = 0.11), indicating that RO did not promote a significant recovery from the after MD state. Finally, we directly compared the recovery produced by MI and RO treatments and found that the after TTX condition had significantly smaller ocular dominance scores than the after RO condition (unpaired *t*-test; *t* = 7.324, d.f. = 12, *p* < 0.001), indicating MI was superior to a comparable period of RO in promoting recovery from a preceding period of MD.

### Ocular axial length

A-scan measurements of ocular axial length from the left and right eyes before and after right eye inactivation are shown in Figure 4. Measurements of axial length obtained from animals in this study were comparable to those made previously from cats that were of similar age (Thorn et al., 1976). Measurements of the left (non-inactivated) eye before inactivation (mean = 16.87 mm; SD = 0.67) were on average not appreciably different from those taken after right eye inactivation (mean = 17.41 mm; SD = 0.74; Figure 4A). While there was a slight increase in average axial length observed for the left eye after inactivation, several animals showed the opposite result, namely a small decrease after inactivation. Similar to the left eye, axial length measurements of the right eye before inactivation (mean = 16.92 mm; SD = 0.72) were not consistently different compared to measurements that were collected from after inactivation (mean = 17.20 mm; SD = 0.59; Figure 4B). To account for the fact that measurements taken after inactivation were from animals at slightly older ages due to the period of inactivation, for each animal we calculated the percentage difference between the left and right eye before and after inactivation (Figure 4C). Before inactivation, the axial length of the left and the right eye were different by an average of less than 0.5%, indicating that no substantial elongation occurred for those animals that received 3 weeks of left eye MD (see Table 2). After inactivation, axial length of the left and right eyes was different only by an average of 1%, indicating that the normal balance in ocular axial length measured between the eyes before inactivation was similar after the period of inactivation. This was confirmed with statistical comparison that showed the percentage difference measured before inactivation was not significantly different from that measured after inactivation (paired *t*-test; *t* = 1.112, d.f. = 8, *p* = 0.298).

**Table 2:** Measurements of axial length and refractive error in all animals examined.

Animal	Inactivation	Axial length (mm)		Refractive error (Diopters)	
		*LE*	*RE*	*LE*	RE
C478	Before	16.75	17.29	+1.5	+0.5
	After	17.78	17.15	+0.375	−0.25
C479	Before	17.17	17.04	0	−0.125
	After	18.39	17.64	+0.75	+0.25
C482	Before	18.51	18.56	+0.75	0
	After	18.26	18.53	+0.875	+0.5
C484	Before	17.05	17.17	+1	+0.5
	After	17.39	16.97	+0.875	+0.5
C485	Before	16.61	16.65		
	After	16.56	16.56		
C486	Before	16.35	16.76	+0.375	+0.625
	After	17.2	17.35	0	+0.75
C491	Before	16.51	16.4		
	After	17.78	16.97		
C492	Before	16.55	16.15		
	After	16.15	16.88		
C493	Before	16.36	16.3		
	After	17.14	16.72		

All TTX injections were made into the right eye. LE indicates measurements from the left eye; RE indicates measurements from the right eye.

### Refractive error

Measurements of refractive error were made by an orthoptist applying objective retinoscopy (Figure 5A) separately for the left and right eyes, and for each animal before and after inactivation of the right eye. In the left eye (Figure 5B), the measured refractive error before inactivation (mean = +0.725 diopters; SD = 0.5 diopters) was similar to that measured after inactivation (mean = +0.575 diopters; SD = 0.4 diopters), and measurements were not statistically different after inactivation (paired *t*-test; *t* = 0.488, d.f. = 4, *p* = 0.65). Similar results were obtained for the right eye (Figure 5C), which was inactivated with microinjection of TTX. The refractive error of the right eye before inactivation (mean = +0.3 diopters; SD = 0.3 diopters) was not appreciably different from what was measured after inactivation (mean = +0.35 diopters; SD = 0.4 diopters), and these before and after measurements were not statistically different (paired *t*-test; *t* = 0.228, d.f. = 4, *p* = 0.83). Therefore, MI did not alter the refractive error of either eye.

### Body weight

**Figure 6:**
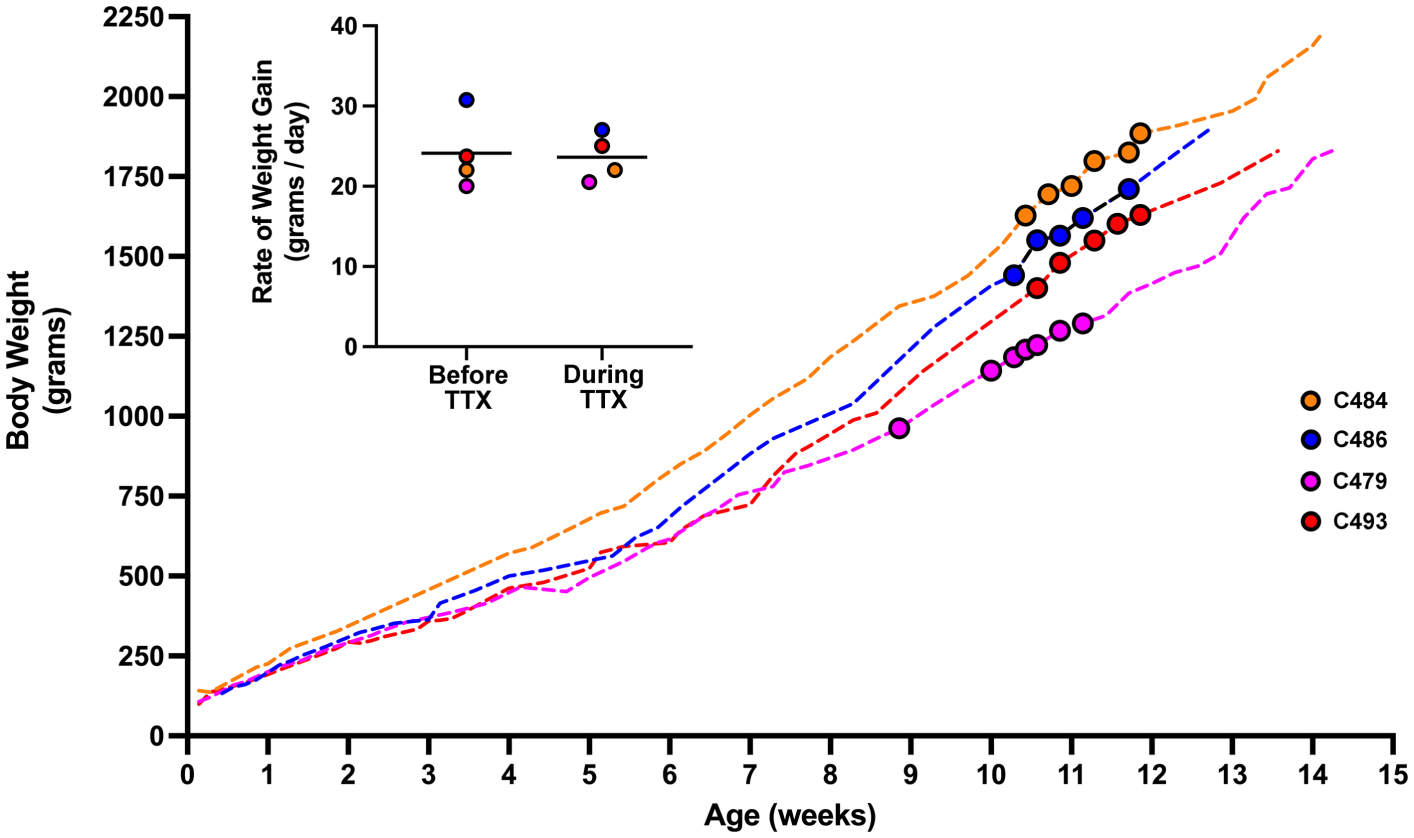
Body weight profile from cats that received MI. Measurements of body weight were made from four animals across the duration of the study. The weight profile for each animal is depicted by a dashed-colored line. The period in development during which MI occurred is highlighted with colored circles. Rate of growth was calculated from weight measurements obtained 10 days prior to MI, and for the duration of MI (inset figure). The rate of growth before and during TTX inactivation were not different. For animal C479, 5 days of binocular vision was provided after the first injection of TTX after which it received four additional injections each separated by 48 h. Our calculation of rate of growth for C479 covered the duration from the first injection until 48 h after the last one. Although consecutive TTX injections were made 48 h apart, measurements of body weight did not necessarily adhere to the same schedule and were sometimes taken more frequently than every 48 h.

As a way of assessing the general health of animals that received MI, we longitudinally tracked the body weight of four cats that received 10 days of MI (Figure 6). If the period of MI was associated with systemic toxicity and illness, we reasoned this might be reflected as a decrease in body weight over the course of inactivation. Leading up to the period of MI, animals exhibited a body weight profile that was comparable to normal (DiGangi et al., 2020; Opsomer et al., 2022). During the 10-day period of inactivation, the growth profile of animals was not disturbed from its developmental trajectory, and animals continued to show a progressive weight gain that was indistinguishable from measurements leading up to the period of inactivation. We quantified the impact of MI by comparing the rate of growth calculated for the 10 days immediately prior to MI, with the growth rate calculated for the duration of MI (Figure 6, inset). The rate of growth before inactivation (mean = 24.1 g per day; SD = 4.6 g per day) and the rate of growth during inactivation (mean = 23.6 g per day; SD = 2.9 g per day) were not significantly different from each other (*t* = 0.435, d.f. = 3, *p* = 0.692). These results indicate that the period of MI did not alter the normal course of weight gain, and implies that monocular microinjection of TTX does not associate with a decline in general health.

## Discussion

This study began with a comparison of the efficacy of MI and RO to promote recovery from the physiologic effects of a long-term MD. Results were clear in showing that following 3 weeks of MD starting at the critical period peak, inactivation of the non-deprived eye for up to 10 days produced superior recovery relative to a comparable duration of RO. Despite the potency of inactivation to promote physiological recovery in visual cortex, measurements of ocular axial length and refractive error for the inactivated eye were not altered by the period of MI, indicating that form-deprivation myopia did not develop. The body weight of a subset of animals was tracked over the course of this study as a measure of general health and well-being during the episode of inactivation. The weight gain profile of animals was not altered by the period of MI, and the rate of growth during the period of MI was not different from the rate of growth before it was started.

The development and deployment of new treatments for amblyopia using animal models are facilitated by a direct comparison of its efficacy with that of conventional therapy. In this study, we showed that after a long-term MD, brief retinal inactivation with microinjection of TTX into the dominant eye restored VEP power that was diminished for the originally deprived eye. Following a TTX washout period, VEPs serving the inactivated eye returned to normal levels and were in balance with VEPs elicited from the other eye. In contrast, occluding the dominant eye after long-term MD did not produce significant recovery of deprived-eye VEP power. This result is consistent with anatomical findings in cats subjected to long-term MD that showed greater recovery of dLGN deprived-eye neuron soma size after inactivation of the dominant eye compared to an equivalent period of RO (Duffy et al., 2018). The absence of significant recovery after RO in the current study is also consistent with past studies that demonstrate diminished potential for reversal from MD at similar ages, and for comparable or even longer durations of RO (Blakemore and Van Sluyters, 1974; Movshon, 1976). After long-term MD in mice, the attenuation of deprived-eye VEPs in V1 were also restored to normal levels after brief inactivation of the dominant eye but similar recovery did not occur after RO (Fong et al., 2021). Nevertheless, because we only examined animals after 10 days of RO, we cannot rule out the possibility that a longer duration of RO would have produced more recovery than what was measured in this study.

The superior efficacy of MI to elicit recovery from the effects of MD may derive from its ability to attenuate the level of activity in V1 and consequently lower the threshold for potentiation of deprived-eye synapses following a sliding threshold model of Hebbian plasticity (Bienenstock et al., 1982; Clothiaux et al., 1991; Fong et al., 2021; Leet et al., 2022). Exposure of mice and rats to complete darkness promotes a shift in the threshold for synaptic strengthening (Kirkwood et al., 1996; Philpot et al., 2003), which may underlie the efficacy of darkness to remediate the effects of MD in rats (He et al., 2007) and cats (Duffy and Mitchell, 2013; Gotou et al., 2021). It is conceivable that a shift in the plasticity threshold may be of greater magnitude following MI compared to darkness because MI eliminates output from the retina whereas darkness eliminates only visually-driven activity. Residual activity driven by the weakened formerly deprived (amblyopic) eye during the period of MI may bootstrap its synaptic recovery, which would not occur with darkness or binocular retinal inactivation. Lowering the threshold for synaptic potentiation induced by MI may align synergistically with the residual activity driven by the originally deprived eye to promote better recovery outcomes compared to an equal period of RO, darkness, or binocular retinal inactivation (Duffy et al., 2018; Holman et al., 2018).

The recovery of VEPs observed after MI may also involve an alteration of inhibition that could relieve an imbalance in interocular perceptual suppression thought to contribute to the deficits in vision linked to amblyopia (Harrad and Hess, 1992; Sengpiel et al., 2006; Li et al., 2011; Hess and Thompson, 2015; Mukerji et al., 2022). The shift in interocular suppression may originate from a modification of the number or size of inhibitory synapses that follow a period of MD (Chen et al., 2011; van Versendaal et al., 2012), and which, along with a reduction in inhibitory drive (Kuhlman et al., 2013), may produce a weakening of inhibitory synapses serving the deprived eye. Indeed, pharmacological experiments implicating GABAergic mechanisms in the shift of ocular dominance produced by MD have demonstrated that administration of bicuculline, a gamma-aminobutyric acid (GABA) antagonist, promotes some restoration of cortical responses elicited from the deprived eye presumably by alleviating the suppressive effect of the non-deprived eye (Duffy et al., 1976; Burchfiel and Duffy, 1981). This suggests that imbalanced interocular suppression contributes to the development of deficits in the deprived eye, and raises the possibility that recovery could occur through a restoration of normal balanced inhibition (Hess and Thompson, 2015). The decrease in cortical activity produced by MI that follows a period MD may shift the modification threshold to favor weakening of inhibitory synapses (Keck et al., 2017), which could reduce inhibitory drive onto deprived excitatory cells. A sustained relief from dominant eye suppression of the deprived eye may develop from long-term depression of inhibitory responses (iLTD) that consolidate the weakening of inhibitory synapses modulating the activity of deprived-eye excitatory neurons (Jiang et al., 2010).

Measurements related to ocular axial length and refractive error indicated that there was not a significant myopic shift over the period of MI examined in this study. Clinically meaningful myopia, referring to a myopic refractive error requiring correction, was not measured in either eye after the period of inactivation. Many of the animals in this study received a period of MD before the fellow eye was inactivated, raising a possible concern that the balanced axial length observed after MI was the result of aberrant and balanced change to both eyes. We believe this is unlikely for two reasons. First, animals that received MD showed a 1% difference in measured axial length between the eyes after the period of MD, and the deprived eye was not consistently the longer of the two (Table 2). Second, measurements of refractive error made after MD revealed a slight hyperopia for the deprived eye (+0.6 diopters), and this was largely unchanged after inactivation of the fellow eye (+0.5 diopters). This measured hyperopia in our study is consistent with previous measurements from normal light-reared cats that were also shown to be slightly hypermetropic (Belkin et al., 1977; Smith et al., 1980). In aggregate, results from this study support previous reports that MD in cats does not consistently produce a significant myopia in the deprived eye (Gollender and Thorn, 1979; Nathan et al., 1984). This variable myopic response suggests that cats may not be an ideal animal model to investigate whether inactivation promotes development of form-deprivation myopia due to an inherent resistance to the impairment. For this reason, we cannot rule out the possibility that MI administered in other species will alter refraction error for the affected eye. Therefore, it will be important to investigate whether other species with a comparable visual system to human exhibit susceptibility to form-deprivation myopia following brief MI.

The duration of MI examined in this study was short (approximately 10 days), raising the possibility that a longer duration of inactivation may have produced a myopic shift for the treated eye. Notwithstanding this possibility, it has been shown that 12 continuous days of MI in tree shews starting at about 5 weeks of age does not produce differences in refractive error, nor did MI produce a difference in the anterior segment depth, or in the corneal radius measured between the eyes (Norton et al., 1994). These results from tree shews are congruent with our result in cats showing no ocular elongation after MI. Interestingly, when the inactivated eye of tree shrews was also subjected to lid closure during the period of MI, a significant myopia developed in the deprived eye (Norton et al., 1994). Collectively these findings suggest that the ganglion cell activity, which is a primary target of TTX inactivation, does not carry the myopia producing message. It is noteworthy that following 12 days of inactivation in tree shews, the vitreous chamber depth of the injected eye was slightly shorter than the fellow eye, which was attributed to the scleral puncture rather than to the inactivation because saline-injected control eyes exhibited a similar small effect (Norton et al., 1994). In the current study, we did not observe that the injected eye was consistently shorter than the fellow eye.

That susceptibility to experimental myopia is higher when visual manipulations begin at younger ages (Troilo and Nickla, 2005); it is possible that animals in the current study were subjected to MI at an age when susceptibility to myopia had waned or past. Although this cannot be ruled out as a possibility and will need to be examined, it is worth noting that monkeys maintain a susceptibility to the effects of visual form deprivation on eye growth and refractive error even into maturity, albeit with a reduced response (Smith et al., 1999; Troilo et al., 2000).

Limitations on conventional therapy for amblyopia raise the importance of investigating novel approaches that may provide superior recovery outcomes. The mainstay treatment for amblyopia, patching, has been in use for centuries (Hoyt, 2015). However, its efficacy to promote recovery is hindered by issues related to the lack of treatment compliance that can be magnified by a requirement for months-long treatment durations (Stewart et al., 2003), recurrence of amblyopia after treatment is complete (Jia et al., 2022), and a transient period in early development when patching can be effective (Holmes and Levi, 2018). The development of a novel therapy that aims to maximize the extent and efficiency of recovery of the impaired eye without compromising ocular health, could offer a significant advancement over available alternatives. Although future studies will assess additional characteristics related to the safe use of brief MI as a means of reversing the effects of visual deprivation, the current set of results demonstrate that MI promotes superior recovery from MD compared to RO, and this is achieved without change to the refractive error or axial length of the inactivated eye. That there was no alteration in weight gain during the period of MI indicates that administration of intravitreal TTX at the doses applied does not impact the general health of animals, and raises the intriguing possibility that retinal inactivation offers a safe and effective alternative to available therapies for amblyopia.

## Data availability statement

The raw data supporting the conclusions of this article will be made available by the authors, without undue reservation.

## Ethics statement

The animal study was reviewed and approved in accordance with the standards of the Canadian Council on Animal Care.

## Author contributions

NC, M-fF, and KRD: experimental design, software development, data collection on physiology experiments, and data analysis. MH, ND, and KRD: experimental design and data collection on the myopia experiments. KRD: figure composition and first manuscript draft. All authors contributed to the article and approved the submitted version.

## Funding

This research was funded by grants from the Natural Sciences and Engineering Research Council of Canada (#RGPIN-2021-02798 to KRD; #RGPIN-2015-06761 to NC) and the Canadian Institutes of Health Research (#468904) to KRD.

## Conflict of interest

The authors declare that the research conducted for this study has no commercial or financial relationships that could be viewed as a conflict of interest.

## Publisher’s note

All claims expressed in this article are solely those of the authors and do not necessarily represent those of their affiliated organizations, or those of the publisher, the editors and the reviewers. Any product that may be evaluated in this article, or claim that may be made by its manufacturer, is not guaranteed or endorsed by the publisher.
